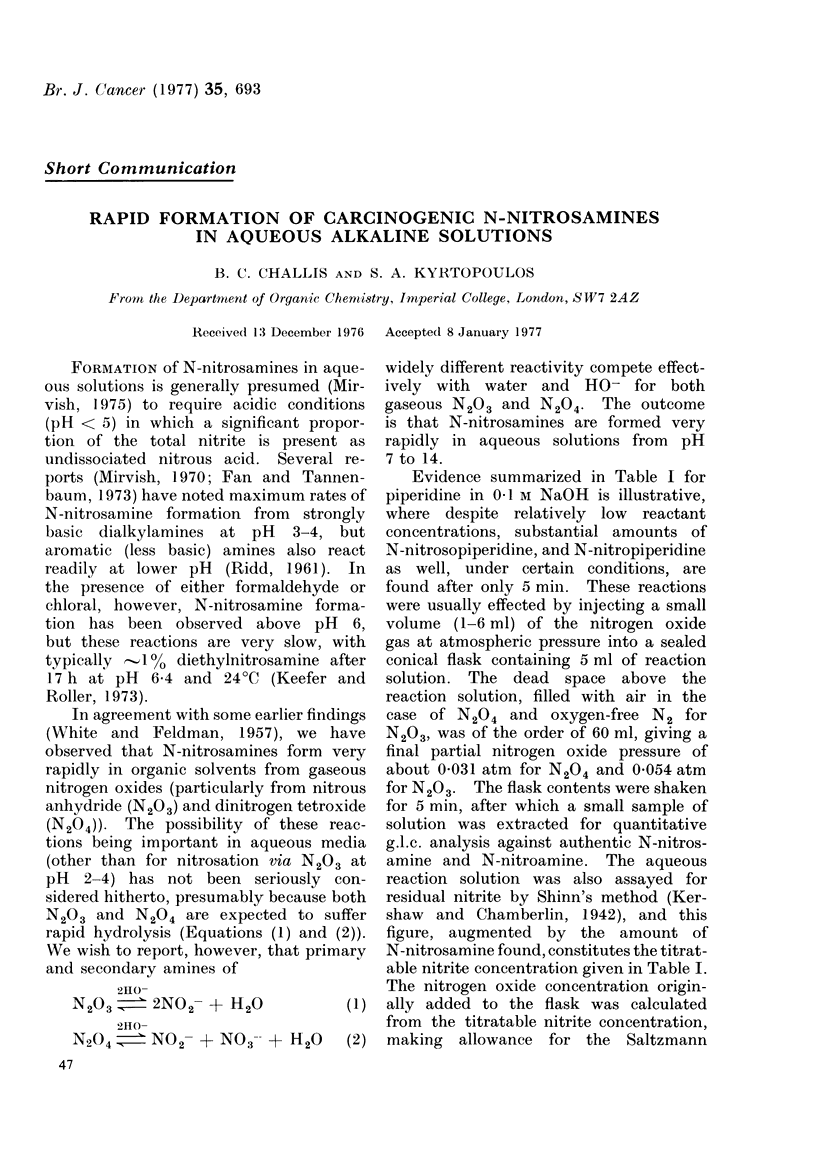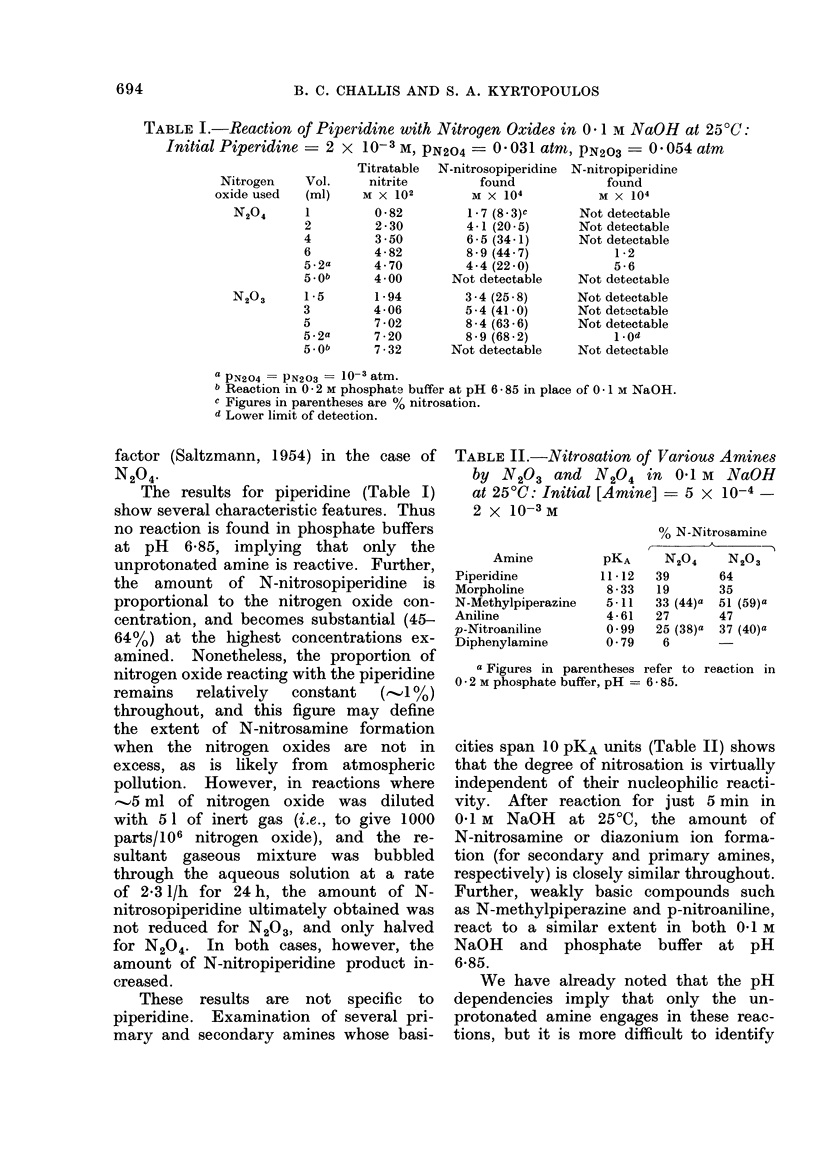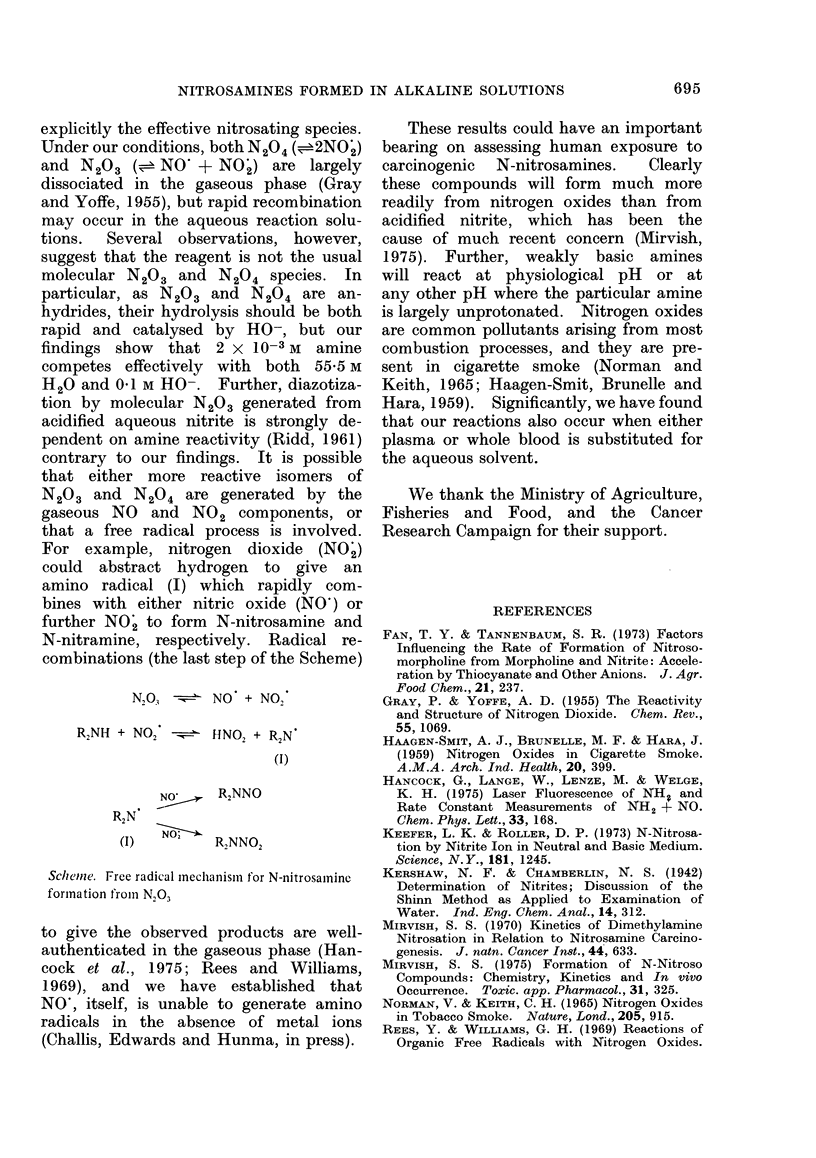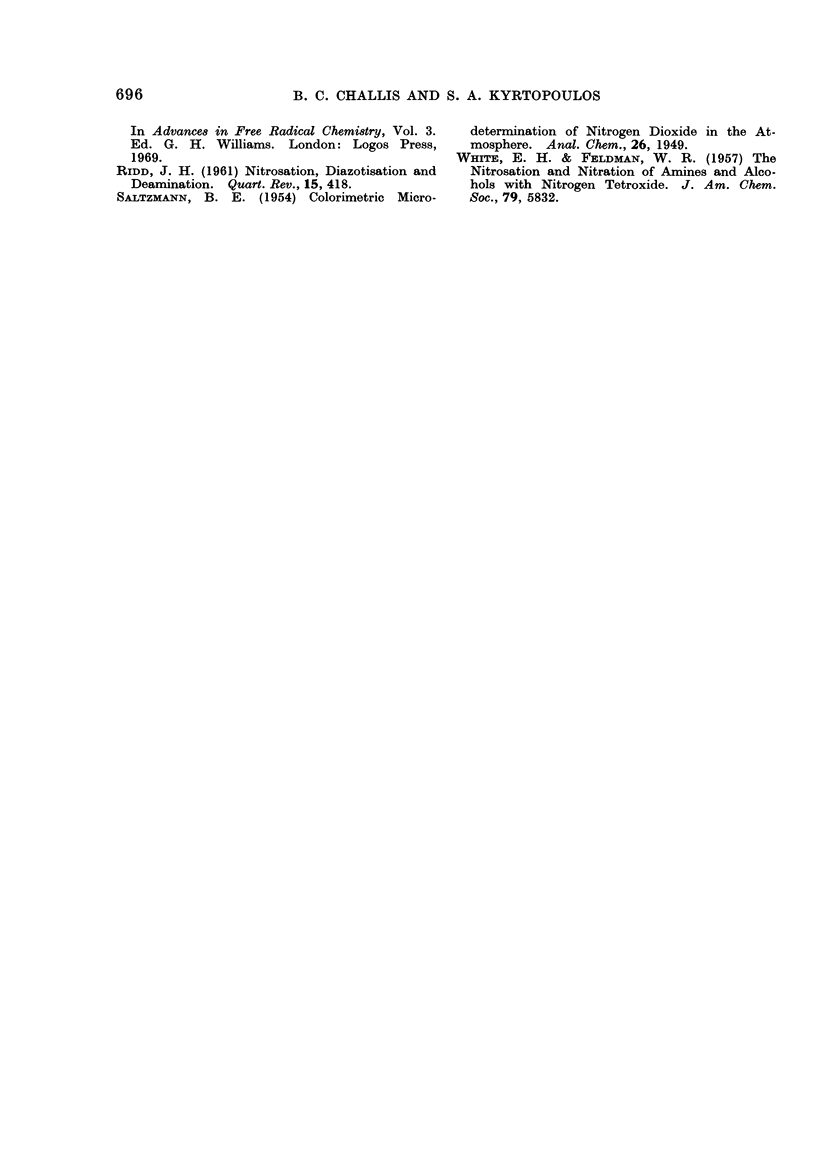# Rapid formation of carcinogenic N-nitrosamines in aqueous alkaline solutions.

**DOI:** 10.1038/bjc.1977.106

**Published:** 1977-05

**Authors:** B. C. Challis, S. A. Kyrtopoulos


Br. J. Cancer (1977) 35, 693

### Short Communication

### RAPID FORMATION OF CARCINOGENIC N-NITROSAMINES IN AQUEOUS ALKALINE SOLUTIONS

B. C. CHALLIS AND S. A. KYRTOPOULOS

*From the Department of Organic Chemistry, Imperial College, London, SW7 2AZ*

Received 13 December 1976 Accepted 8 January 1977

FORMATION of N-nitrosamines in aqueous solutions is generally presumed (Mirvish, 1975) to require acidic conditions ($pH < 5$) in which a significant proportion of the total nitrite is present as undissociated nitrous acid. Several reports (Mirvish, 1970; Fan and Tannenbaum, 1973) have noted maximum rates of N-nitrosamine formation from strongly basic dialkylamines at pH 3–4, but aromatic (less basic) amines also react readily at lower pH (Ridd, 1961). In the presence of either formaldehyde or chloral, however, N-nitrosamine formation has been observed above pH 6, but these reactions are very slow, with typically ~1% diethylnitrosamine after 17 h at pH 6·4 and 24°C (Keefer and Roller, 1973).

In agreement with some earlier findings (White and Feldman, 1957), we have observed that N-nitrosamines form very rapidly in organic solvents from gaseous nitrogen oxides (particularly from nitrous anhydride ($N_2O_3$) and dinitrogen tetroxide ($N_2O_4$)). The possibility of these reactions being important in aqueous media (other than for nitrosation *via* $N_2O_3$ at pH 2–4) has not been seriously considered hitherto, presumably because both $N_2O_3$ and $N_2O_4$ are expected to suffer rapid hydrolysis (Equations (1) and (2)). We wish to report, however, that primary and secondary amines of

$$N_2O_3 \xrightleftharpoons{2HO^-} 2NO_2^- + H_2O \qquad (1)$$

$$N_2O_4 \xrightleftharpoons{2HO^-} NO_2^- + NO_3^- + H_2O \qquad (2)$$

widely different reactivity compete effectively with water and $HO^-$ for both gaseous $N_2O_3$ and $N_2O_4$. The outcome is that N-nitrosamines are formed very rapidly in aqueous solutions from pH 7 to 14.

Evidence summarized in Table I for piperidine in 0·1 M NaOH is illustrative, where despite relatively low reactant concentrations, substantial amounts of N-nitrosopiperidine, and N-nitropiperidine as well, under certain conditions, are found after only 5 min. These reactions were usually effected by injecting a small volume (1–6 ml) of the nitrogen oxide gas at atmospheric pressure into a sealed conical flask containing 5 ml of reaction solution. The dead space above the reaction solution, filled with air in the case of $N_2O_4$ and oxygen-free $N_2$ for $N_2O_3$, was of the order of 60 ml, giving a final partial nitrogen oxide pressure of about 0·031 atm for $N_2O_4$ and 0·054 atm for $N_2O_3$. The flask contents were shaken for 5 min, after which a small sample of solution was extracted for quantitative g.l.c. analysis against authentic N-nitrosamine and N-nitroamine. The aqueous reaction solution was also assayed for residual nitrite by Shinn's method (Kershaw and Chamberlin, 1942), and this figure, augmented by the amount of N-nitrosamine found, constitutes the titratable nitrite concentration given in Table I. The nitrogen oxide concentration originally added to the flask was calculated from the titratable nitrite concentration, making allowance for the Saltzmann

TABLE I.—*Reaction of Piperidine with Nitrogen Oxides in* 0·1 M *NaOH at* 25°C: *Initial Piperidine* = $2 \times 10^{-3}$ M, $p_{N_2O_4}$ = 0·031 *atm*, $p_{N_2O_3}$ = 0·054 *atm*

| Nitrogen oxide used | Vol. (ml) | Titratable nitrite M × $10^2$ | N-nitrosopiperidine found M × $10^4$ | N-nitropiperidine found M × $10^4$ |
|---|---|---|---|---|
| $N_2O_4$ | 1 | 0·82 | 1·7 (8·3)[c] | Not detectable |
| | 2 | 2·30 | 4·1 (20·5) | Not detectable |
| | 4 | 3·50 | 6·5 (34·1) | Not detectable |
| | 6 | 4·82 | 8·9 (44·7) | 1·2 |
| | 5·2[a] | 4·70 | 4·4 (22·0) | 5·6 |
| | 5·0[b] | 4·00 | Not detectable | Not detectable |
| $N_2O_3$ | 1·5 | 1·94 | 3·4 (25·8) | Not detectable |
| | 3 | 4·06 | 5·4 (41·0) | Not detectable |
| | 5 | 7·02 | 8·4 (63·6) | Not detectable |
| | 5·2[a] | 7·20 | 8·9 (68·2) | 1·0[d] |
| | 5·0[b] | 7·32 | Not detectable | Not detectable |

[a] $p_{N_2O_4} = p_{N_2O_3} = 10^{-3}$ atm.
[b] Reaction in 0·2 M phosphate buffer at pH 6·85 in place of 0·1 M NaOH.
[c] Figures in parentheses are % nitrosation.
[d] Lower limit of detection.

factor (Saltzmann, 1954) in the case of $N_2O_4$.

The results for piperidine (Table I) show several characteristic features. Thus no reaction is found in phosphate buffers at pH 6·85, implying that only the unprotonated amine is reactive. Further, the amount of N-nitrosopiperidine is proportional to the nitrogen oxide concentration, and becomes substantial (45–64%) at the highest concentrations examined. Nonetheless, the proportion of nitrogen oxide reacting with the piperidine remains relatively constant (~1%) throughout, and this figure may define the extent of N-nitrosamine formation when the nitrogen oxides are not in excess, as is likely from atmospheric pollution. However, in reactions where ~5 ml of nitrogen oxide was diluted with 5 l of inert gas (*i.e.*, to give 1000 parts/$10^6$ nitrogen oxide), and the resultant gaseous mixture was bubbled through the aqueous solution at a rate of 2·3 l/h for 24 h, the amount of N-nitrosopiperidine ultimately obtained was not reduced for $N_2O_3$, and only halved for $N_2O_4$. In both cases, however, the amount of N-nitropiperidine product increased.

These results are not specific to piperidine. Examination of several primary and secondary amines whose basicities span 10 $pK_A$ units (Table II) shows that the degree of nitrosation is virtually independent of their nucleophilic reactivity. After reaction for just 5 min in 0·1 M NaOH at 25°C, the amount of N-nitrosamine or diazonium ion formation (for secondary and primary amines, respectively) is closely similar throughout. Further, weakly basic compounds such as N-methylpiperazine and p-nitroaniline, react to a similar extent in both 0·1 M NaOH and phosphate buffer at pH 6·85.

TABLE II.—*Nitrosation of Various Amines by* $N_2O_3$ *and* $N_2O_4$ *in* 0·1 M *NaOH at* 25°C: *Initial* [*Amine*] = $5 \times 10^{-4}$ – $2 \times 10^{-3}$ M

| Amine | $pK_A$ | % N-Nitrosamine $N_2O_4$ | % N-Nitrosamine $N_2O_3$ |
|---|---|---|---|
| Piperidine | 11·12 | 39 | 64 |
| Morpholine | 8·33 | 19 | 35 |
| N-Methylpiperazine | 5·11 | 33 (44)[a] | 51 (59)[a] |
| Aniline | 4·61 | 27 | 47 |
| *p*-Nitroaniline | 0·99 | 25 (38)[a] | 37 (40)[a] |
| Diphenylamine | 0·79 | 6 | — |

[a] Figures in parentheses refer to reaction in 0·2 M phosphate buffer, pH = 6·85.

We have already noted that the pH dependencies imply that only the unprotonated amine engages in these reactions, but it is more difficult to identify explicitly the effective nitrosating species. Under our conditions, both $N_2O_4$ ($\rightleftharpoons 2NO_2^{\cdot}$) and $N_2O_3$ ($\rightleftharpoons NO^{\cdot} + NO_2^{\cdot}$) are largely dissociated in the gaseous phase (Gray and Yoffe, 1955), but rapid recombination may occur in the aqueous reaction solutions. Several observations, however, suggest that the reagent is not the usual molecular $N_2O_3$ and $N_2O_4$ species. In particular, as $N_2O_3$ and $N_2O_4$ are anhydrides, their hydrolysis should be both rapid and catalysed by $HO^-$, but our findings show that $2 \times 10^{-3}$ M amine competes effectively with both 55·5 M $H_2O$ and 0·1 M $HO^-$. Further, diazotization by molecular $N_2O_3$ generated from acidified aqueous nitrite is strongly dependent on amine reactivity (Ridd, 1961) contrary to our findings. It is possible that either more reactive isomers of $N_2O_3$ and $N_2O_4$ are generated by the gaseous NO and $NO_2$ components, or that a free radical process is involved. For example, nitrogen dioxide ($NO_2^{\cdot}$) could abstract hydrogen to give an amino radical (I) which rapidly combines with either nitric oxide ($NO^{\cdot}$) or further $NO_2^{\cdot}$ to form N-nitrosamine and N-nitramine, respectively. Radical recombinations (the last step of the Scheme)

*Scheme.* Free radical mechanism for N-nitrosamine formation from $N_2O_3$

to give the observed products are well-authenticated in the gaseous phase (Hancock *et al.*, 1975; Rees and Williams, 1969), and we have established that $NO^{\cdot}$, itself, is unable to generate amino radicals in the absence of metal ions (Challis, Edwards and Hunma, in press).

These results could have an important bearing on assessing human exposure to carcinogenic N-nitrosamines. Clearly these compounds will form much more readily from nitrogen oxides than from acidified nitrite, which has been the cause of much recent concern (Mirvish, 1975). Further, weakly basic amines will react at physiological pH or at any other pH where the particular amine is largely unprotonated. Nitrogen oxides are common pollutants arising from most combustion processes, and they are present in cigarette smoke (Norman and Keith, 1965; Haagen-Smit, Brunelle and Hara, 1959). Significantly, we have found that our reactions also occur when either plasma or whole blood is substituted for the aqueous solvent.

We thank the Ministry of Agriculture, Fisheries and Food, and the Cancer Research Campaign for their support.

### REFERENCES

Fan, T. Y. & Tannenbaum, S. R. (1973) Factors Influencing the Rate of Formation of Nitrosomorpholine from Morpholine and Nitrite: Acceleration by Thiocyanate and Other Anions. *J. Agr. Food Chem.*, **21**, 237.

Gray, P. & Yoffe, A. D. (1955) The Reactivity and Structure of Nitrogen Dioxide. *Chem. Rev.*, **55**, 1069.

Haagen-Smit, A. J., Brunelle, M. F. & Hara, J. (1959) Nitrogen Oxides in Cigarette Smoke. *A.M.A. Arch. Ind. Health*, **20**, 399.

Hancock, G., Lange, W., Lenze, M. & Welge, K. H. (1975) Laser Fluorescence of $NH_2$ and Rate Constant Measurements of $NH_2 + NO$. *Chem. Phys. Lett.*, **33**, 168.

Keefer, L. K. & Roller, D. P. (1973) N-Nitrosation by Nitrite Ion in Neutral and Basic Medium. *Science, N.Y.*, **181**, 1245.

Kershaw, N. F. & Chamberlin, N. S. (1942) Determination of Nitrites; Discussion of the Shinn Method as Applied to Examination of Water. *Ind. Eng. Chem. Anal.*, **14**, 312.

Mirvish, S. S. (1970) Kinetics of Dimethylamine Nitrosation in Relation to Nitrosamine Carcinogenesis. *J. natn. Cancer Inst.*, **44**, 633.

Mirvish, S. S. (1975) Formation of N-Nitroso Compounds: Chemistry, Kinetics and *In vivo* Occurrence. *Toxic. app. Pharmacol.*, **31**, 325.

Norman, V. & Keith, C. H. (1965) Nitrogen Oxides in Tobacco Smoke. *Nature, Lond.*, **205**, 915.

Rees, Y. & Williams, G. H. (1969) Reactions of Organic Free Radicals with Nitrogen Oxides.

In *Advances in Free Radical Chemistry*, Vol. 3. Ed. G. H. Williams. London: Logos Press, 1969.

RIDD, J. H. (1961) Nitrosation, Diazotisation and Deamination. *Quart. Rev.*, **15**, 418.

SALTZMANN, B. E. (1954) Colorimetric Microdetermination of Nitrogen Dioxide in the Atmosphere. *Anal. Chem.*, **26**, 1949.

WHITE, E. H. & FELDMAN, W. R. (1957) The Nitrosation and Nitration of Amines and Alcohols with Nitrogen Tetroxide. *J. Am. Chem. Soc.*, **79**, 5832.